# Different patient case mix by applying the 2003 SCCM/ESICM/ACCP/ATS/SIS sepsis definitions instead of the 1992 ACCP/SCCM sepsis definitions in surgical patients: a retrospective observational study

**DOI:** 10.1186/1472-6947-9-25

**Published:** 2009-05-18

**Authors:** Manfred Weiss, Markus Huber-Lang, Michael Taenzer, Karl Traeger, Juergen Altherr, Martina Kron, Birgit Hay, Marion Schneider

**Affiliations:** 1 Department of Anaesthesiology, University Hospital Ulm, Steinhoevelstr. 9, 89075 Ulm, Germany; 2 Department of Traumatology, Hand, Plastic, and Reconstructive Surgery, University Hospital Ulm, Steinhoevelstr. 9, 89075 Ulm, Germany; 3 Institute of Biometrics, University of Ulm, Schwabstr. 13, 89075 Ulm, Germany; 4 Department of Experimental Anaesthesiology, University Hospital Ulm, Steinhoevelstr. 9, 89075 Ulm, Germany

## Abstract

**Background:**

Revised consensus sepsis definitions have been published in 2003. The present study was performed to compare the prevalence of different stages of sepsis and ICU mortality rates and find out the case mix within the same collective of postoperative/posttraumatic patients applying either the original 1992 ACCP/SCCM or the revised 2003 SCCM/ESICM/ACCP/ATS/SIS sepsis definitions.

**Methods:**

Retrospective observational single-centre study in surgical critically ill patients admitted to an University adult ICU. From 01/2007 to 12/2007, 742 patients were surveyed daily computer-assisted with respect to different stages of sepsis.

**Results:**

Within the same patient collective, applying the 2003 definitions instead of the 1992 definitions, prevalence of severe sepsis (61 vs. 56) and septic shock (205 vs. 162) was higher (p < 0.001). In patients with septic shock according to the 2003 definitions, mortality rate of 22% was lower than that of 27%, when the 1992 definitions were used. Risk of death was increased for those patients classified to be in septic shock with any of the definitions (OR 6.5, p = 0.001). Sensitivity to predict deaths was slightly higher with the 2003 definitions (92%) than with the 1992 definitions (88%), and specificity was lower (31% vs. 49%).

**Conclusion:**

The prevalence and mortality rates of various sepsis severity stages differ if defined by the 1992 or the 2003 definitions. Thus, transferring conclusions drawn from data sets regarding severity of sepsis generated with the 1992 definitions to the same population applying the 2003 definitions may be misleading. The 1992 definitions may under-classify patients with severe sepsis.

## Background

Prior to 1987, none of the sepsis studies utilized standards for inclusion criteria [1]. Since the 1992 American College of Chest Physicians/Society of Critical Care Medicine (ACCP/SCCM) consensus conference on sepsis definitions [2], predefined sepsis criteria for patient enrolment in clinical trials and markers of organ dysfunction have been increasingly applied [1]. Comparing 176 trials from 1993 to 2001 after the ACCP/SCCM consensus conference with 57 trials from 1976 to 1992, revealed increasing use of standards for inclusion criteria in 65% vs. 11%, respectively, in most cases (69%) the ACCP/SCCM consensus conference definitions [1]. Uniformity of inclusion criteria is a prerequisite for comparability of sepsis studies. In 2001, the original 1992 ACCP/SCCM sepsis definitions [2] were modified to reflect the extended understanding of the pathophysiology of the systemic inflammatory response syndrome (SIRS) and sepsis syndrome at that time. The result was the 2003 revised Society of Critical Care Medicine/European Society of Critical Care Medicine/American College of Chest Physicians/American Thoracic Society, Surgical Infection Society (SCCM/ESICM/ACCP/ATS/SIS) sepsis definition [3,4]. However, due to its complexity, the latter definition is not widely used, and papers and current studies still apply to the 1992 definitions.

In the 2003 sepsis definitions, it was stated that the diagnostic criteria for infection or systemic inflammation should be broadly useful for both clinicians caring for patients at the bedside and researchers designing observational studies and clinical trials to improve the understanding of sepsis and its treatment. An extended list of possible signs of the systemic response was included in the 2003 definitions to better reflect the reality at the bedside, especially, of how physicians diagnose sepsis in practice, regarding general, inflammatory, hemodynamic, organ dysfunction, and tissue perfusion variables. In Table 1, the diagnostic criteria for sepsis and organ dysfunction variables in the 1992 and 2003 definitions are specified and compared. Cut-off values changed from the 1992 definitions to the 2003 definitions, such as fever (from > 38.0°C to > 38.3°C) and tachypnea (from > 20 to > 30 breaths/min). Furthermore, in the 1990s, it was recommended to define organ failure by applying the Sequential Organ Failure Assessment (SOFA) score with greater than two points in one organ system [5]. In contrast to the 1992 definitions, the 2003 definitions provide clear cut-off values for organ dysfunctions. The use of the word "some" of the diagnostic criteria in the 2003 definitions instead of "two or more out of four" (temperature, heart rate, respiratory rate, and white blood cell count (WBC)) in the 1992 definitions, was introduced to facilitate a clinical diagnosis at the bedside. Once severe sepsis and septic shock are diagnosed, this may lead to far-reaching consequences since extensive management and treatment guidelines have been published for this issue [6,7].

**Table 1:** Diagnostic criteria and organ dysfunction variables within the 1992 ACCP/SCCM and the 2003 SCCM/ESICM/ACCP/ATS/SIS sepsis definitions

	ACCP/SCCM 1992	SCCM/ESICM/ACCP/ATS/SIS 2003
**Diagnostic criteria for sepsis**		
		
Infection	+	+
Temperature	<36.0°C and/or >38.0°C	<36.0°C and/or >38.3°C
Heart rate	>90	>90
Respiratory rate	>20	>30
White blood cell count (WBC)	>12,000/μl and/or <4,000/μl and/or >10% immature	>12,000/μl and/or <4,000/μl and/or >10% immature
Altered mental status	n. a.	+
Significant edema	n. a.	+
Positive fluid balance	n. a.	>20 ml/kg over 24 hrs
Hyperglycemia in absence of diabetes	n. a.	>120 mg/dl or 7.7 mmol/l
Plasma C-reactive protein	n. a.	>2 SD above normal
Plasma procalcitonin	n. a.	>2 SD above normal
SvO_2_	n. a.	>70%
Cardiac index	n. a.	>3.5 l/min/m^2^
		
**Organ dysfunction variables**		
		
Altered mental status	+	n. a.
Lactic acidosis	+	n. a.
Acute oliguria	<500 ml/24 h	<0.5 ml/kg/h or 45 mmol/l >2 h
Hypoperfusion or hypotension	+	+
SOFA score	>2	n. a.
Creatinine	n. a. >3.5 mg/dl or 300 μmol/l*	n. a.
Creatinin increase	n. a.	>0.5 mg/dl or 43 μmol/l
Arterial hypoxemia	n. a. PaO_2_/FiO_2 _<= 200 with respiratory support*	PaO_2_/FiO_2 _< 300
Coagulation abnormalities	n. a.	INR > 1.5 or aPTT > 60s
Ileus (absent bowel sounds)	n. a.	+
Thrombocytopenia, platelet count	n. a. <= 50,000/μl*	<100,000/μl
Hyperbilirubinemia, plasma total bilirubin	n. a. >6 mg/dl or 102 μmol/l*	>4 mg/dl or 70 μmol/l
Hyperlactatemia	n. a.	>1 mmol/l

n. a. = not applicable, INR = International Normalized Ratio, PaO_2_/FiO_2 _= partial pressure of arterial oxygen/fraction of inspired oxygen, SOFA = Sequential Organ Failure Assessement, SvO_2 _= mixed venous oxygen saturation, *according to the definition of organ dysfunction with greater than 2 points in the SOFA score.

However, it remains unclear how the changes in the definitions influence the prevalence and mortality rate of SIRS and sepsis in critically ill surgical patients. The 2003 sepsis definitions were updated for clinicians and researchers to facilitate a clinical diagnosis and to improve the understanding, diagnosis and treatment of sepsis. Therefore, the paper will focus on patients with sepsis. The present study was performed to determine how the prevalence of various stages of sepsis and mortality rates differs in the same patient population, if defined by either the original or the revised sepsis criteria. Moreover, it should be addressed whether a switch to the 2003 definitions might increase the number of patients who are expected to benefit from earlier and more appropriate critical care management.

## Methods

### Patients and data collection

The study is in compliance with the Helsinki declaration and was approved by the ethics committee of the University Hospital Ulm. The ethics committee waived informed consent due to the fact that for this study no blood was drawn and no intervention was performed. Thus, no informed consent was obtained from the patients. Patients were admitted to the Anaesthesiolgical ICU of the University Hospital Ulm after major trauma, great vessel, lung, brain or abdominal surgery. All surgical patients admitted to this ICU are routinely computer-assisted surveyed for sepsis, severity of disease (Simplified Acute Physiology Score (SAPS) II) [8] and organ dysfunctions (Sequential Organ Failure Assessment (SOFA) score) [5] on a daily basis by the ICU residents and staff physicians. To improve accuracy and to minimize inconsistencies in medical chart reviews, all residents and staff physicians were trained in the charts before initiation of the study. In addition, cases were selected, variables were defined, and data were entered on a daily base in standardized electronic case report forms leading through the different organ systems and relevant infection parameters. After data entry of different organ systems and infection parameters, the physicians directly received the results of the actual scores, the sepsis/SIRS classification, and, also, a longitudinal overview regarding the whole ICU course. Then, the residents and staff physicians checked, corrected, ascertained and re-ascertained the data and classifications. Monitoring and meetings were performed daily and inter-rater agreement tested. Charts were checked and corrected by the staff physicians before demission of the patients from the ICU and before final evaluation. Data of postoperative/posttraumatic patients admitted to our ICU during a one year period from 01-JAN-2007 until 31-DEC-2007 were analysed.

### Definitions

Sepsis was defined using the original 1992 ACCP/SCCM [2] and the revised 2003 SCCM/ESICM/ACCP/ATS/SIS sepsis definitions [3,4]. In the present study, due to the 1992 definitions, a systemic inflammatory response syndrome (SIRS) was manifested in patients by two or more of the four conditions: temperature, heart rate, respiratory rate, and white blood cell count (WBC) (Table 1). If SIRS was due to a documented infection, patients were classified as sepsis patients. In the present study, applying the 2003 sepsis definitions, SIRS and sepsis was classified as manifested if two or more of eight conditions given in the broadened diagnostic criteria list of possible signs of systemic inflammation were present. These criteria include general variables such as temperature, heart rate, respiratory rate, altered mental status (not applied in the present study), significant edema or positive fluid balance, hyperglycemia, as well as inflammatory variables such as white blood cell count (WBC), plasma C-reactive protein and plasma procalcitonin (not applied in the present study). Severe sepsis was defined as sepsis plus organ dysfunction. Septic shock was defined as severe sepsis plus shock. Severity of sepsis is proposed to increase firstly by association with organ dsyfunctions and secondly by additional shock. As recommended [5], in the present study, applying the 1992 sepsis definitions, organ failure was regarded to be present if patients had lactic acidosis or oliguria, or reached greater than two points in one organ system (lung, coagulation, liver, kidney) using the SOFA score. Greater than two points are reached in the SOFA score for the organ system lung with PaO_2_/FIO_2 _<= 200 with respiratory support, for coagulation with platelets <= 50,000/μl, for liver with bilirubin > 6 mg/dl or 102 μmol/l, for kidney with creatinine > 3.5 mg/dl or > 300 μmol/l or with urine output < 500 ml/day (Table 1). With respect to the 2003 sepsis definitions, organ dysfunctions were defined according to the limitations for organ dysfunction variables and tissue perfusion variables (hyperlactatemia) as presented in Table 1. Septic shock was defined as hypotension despite adequate volume resuscitation, a systolic blood pressure of <= 90 mmHg, or the need of vasopressors to keep blood pressure greater than 90 mmHg. For each patient, the worst degree of sepsis severity during the ICU stay with the 1992 and the 2003 definitions, respectively, was taken for analysis.

### Statistical analyses

ICU mortality rates in different stages of sepsis according to the 1992 and the 2003 sepsis definitions were compared descriptively. The prevalence of severe SIRS and SIRS shock according to the 1992 and the 2003 definitions among patients without infections were compared by McNemar's test. Analogously, the prevalence of severe sepsis and septic shock according to the 1992 and the 2003 definitions were compared among patients with infections. Logistic regression for the outcome death among patients with documented infections was applied to simultaneously assess the impact of the 1992 and the 2003 sepsis definitions. Odds ratios (ORs), with corresponding 95% confidence interval (CI) and p-values are presented. Sensitivity and specificity of the 1992 and 2003 definitions to predict deaths among patients with documented infections were calculated with 95% CI.

## Results

From 01/2007 to 12/2007, 827 postoperative/posttraumatic patients were admitted to the ICU, 765 were surveyed daily using computer-assistance with respect to SIRS and sepsis. Scores with the 1992 and the 2003 definitions were available in 742 patients. Clinical characteristics of the 742 patients were as following. Median age was 66 years (range: 5 months to 100 years; mean +/- SD: 61 +/- 19 years; 23 patients less than 18 years). 252 of 742 patients were female and 490 were male. Median SAPS II was 34 (range: 0 – 97; mean +/- SD: 39 +/- 20). Median SOFA score (due to analgosedation without Glasgow Coma Score, thus, theoretical maximum of 20) was 5 (range: 0 – 18; mean +/- SD: 6 +/- 4). Out of the 742 patients, 184 patients were admitted to our ICU after abdominal surgery, 187 patients after great vessel or lung surgery, 141 patients after major trauma and damage control orthopaedic surgery, 228 patients due to neurosurgery and two patients due to other reasons. Causes of infections were pneumonia, bloodstream infections, intravascular catheter-related infections, intra-abdominal infections, urological infections and surgical wound infections.

### SIRS patients

Among the 742 patients, 460 patients were without clinically or microbiologically documented infections, ranging from no systemic inflammatory response up to SIRS shock with an overall mortality rate of 6%. The prevalence of no SIRS (9 vs. 115) and SIRS (137 vs. 184) was lower with the revised 2003 definitions than with the 1992 definitions (Table 2). However, applying the 2003 definitions instead of the 1992 definitions, the prevalence of severe SIRS (191 vs. 97) and SIRS shock (123 vs. 64) was higher (p < 0.001). Mortality rate in patients with SIRS shock of 15% by use of the 2003 definitions was lower than that of 22% by that of the 1992 definitions (Table 2).

**Table 2:** Prevalence and mortality rates of patients defined by 1992 ACCP/SCCM and 2003 SCCM/ESICM/ACCP/ATS/SIS sepsis definitions.

	ACCP/SCCM 1992			SCCM/ESICM/ACCP/ATS/SIS 2003		
	
Score	Number	Dead	Mortality(%)	Number	Dead	Mortality(%)
No infection, no SIRS	115	0	0	9	1	11
SIRS	184	7	4	137	5	4
Severe SIRS	97	7	7	191	4	2
SIRS shock	64	14	22	123	18	15
						
*Total SIRS group*	*460*	*28*	*6*	*460*	*28*	*6*
						
Infection, no sepsis	13	0	0	0	0	0
Sepsis	51	4	8	16	2	13
Severe sepsis	56	2	4	61	2	3
Septic shock	162	43	27	205	45	22
						
*Total sepsis group*	*282*	*49*	*17*	*282*	*49*	*17*
						
Total patients	742	77	10	742	77	10

SIRS = systemic inflammatory response syndrome, ACCP/SCCM = American College of Chest Physicians/Society of Critical Care Medicine, SCCM/ESICM/ACCP/ATS/SIS = Society of Critical Care Medicine/European Society of Critical Care Medicine/American College of Chest Physicians/American Thoracic Society, Surgical Infection Society.

### Sepsis patients

Among the 742 patients, 282 with clinically or microbiologically proven infections suffered from infections without sepsis up to septic shock, with an overall mortality rate of 17%. The prevalence of infection without sepsis (0 vs. 13) and with sepsis (16 vs. 51) was lower applying the 2003 definitions than the 1992 definitions (Table 2). On the other hand, using the 2003 definitions instead of the 1992 definitions, the prevalence of severe sepsis (61 vs. 56) and septic shock (205 vs. 162) was higher (p < 0.001). Mortality rate in patients with septic shock of 22% applying the 2003 definitions was lower than that of 27% obtained using the 1992 definitions (Table 2).

Among the 282 patients with documented infections, mortality rates of patients classified to be without septic shock in both definitions, were compared with those of patients with septic shock in either or both definitions. Risk of death was profoundly higher in those patients, which were classified to be in septic shock in both definitions compared to those classified to be without septic shock in both definitions (OR 6.5, 95% CI 2.2 – 18.8, p = 0.001, 43 dead, 116 alive). The risk of death in patients classified as septic shock in the 2003 definitions only (OR 0.8, CI 0.1 – 4.5, p = 0.797, 2 dead, 44 alive) and those classified as without septic shock in both definitions (4 dead, 70 alive) was very similar. The risk of death in patients classified as septic shock in the 1992 definitions only, and those classified as without septic shock in the 2003 definitions could not be analysed due to the low number of patients, i.e., none dead and 3 alive.

Sensitivity of predicting deaths in patients with documented infections was slightly higher using the 2003 definitions than the 1992 definitions (92% vs. 88%) while specificity was lower (31% vs. 49%) (Table 3).

**Table 3:** Sensitivity and specificity of predicting deaths in critically ill patients with documented infections

Patients	Predicted deaths/total deaths	Sensitivity	95% CI
Positive in 1992 definitions	43/49	88%	75% – 95%
Positive in 2003 definitions	45/49	92%	80% – 98%
Positive in at least one definition	45/49	92%	80% – 98%
Positive in both definitions	43/49	88%	75% – 95%

**Patients**	**Predicted survivors/total survivors**	**Specificity**	**95% CI**

Negative in 1992 definitions	114/233	49%	42% – 56%
Negative in 2003 definitions	73/233	31%	25% – 38%
Negative in at least one definition	117/233	50%	44% – 57%
Negative in both definitions	70/233	30%	24% – 36%

### Patients with a high risk of death

As shown in Table 2, patients with shock were at a high risk of death. Table 4 summarizes the number of cases with matching classification and those with divergent classification following the 1992 or the 2003 definitions, respectively. Out of the 742 patients, 412 had a matching classification, 21 patients were classified with higher scores using the 1992 definitions as compared to the 2003 definitions, 309 patients had higher scores with the 2003 as compared to the 1992 definitions. Out of the 338 shock patients, 216 had a matching classification, 10 patients were classified with higher scores with the 1992 definitions, and 112 patients with higher scores with the 2003 definitions. In the patients with severe SIRS and sepsis, 140/225 patients were under-classified with the 1992 definitions, whereas 9 with the 2003 definitions. In the subgroup of the 338 shock patients following the 1992 and/or the 2003 definitions, 65 died (19%), and 216 had a common classification. 112/338 shock patients were under-classified with the 1992 definitions, while 10 with the 2003 definitions, only. Out of the 65/338 shock non-survivors, 55 had a matching classification. Eight/65 non-survivors in shock were under-classified with the 1992 definitions, however, two with the 2003 definitions, only. In total, 10/65 non-survivors were misclassified. Six/65 were assigned to the shock group using the 2003 definitions, only.

**Table 4:** Classification of patients defined by 1992 ACCP/SCCM and 2003 SCCM/ESICM/ACCP/ATS/SIS sepsis definitions.

ACCP/SCCM 1992	SCCM/ESICM/ACCP/ATS/SIS 2003	Alive+dead	Alive	Dead	Mortality(%)
**Matching classification 1992 = 2003**					

**Septic shock**	**Septic shock**	159	116	43	**37**
**SIRS shock**	**SIRS shock**	57	45	12	**27**
Severe sepsis	Severe sepsis	34	33	1	3
Severe SIRS	Severe SIRS	72	69	3	4
Sepsis	Sepsis	14	12	2	17
SIRS	SIRS	69	66	3	5
Infection	Infection	0	0	0	0
No infection	No infection	7	7	0	0
***Total shock patients***		***216***	***161***	***55***	***25***
*Total severe patients*		*106*	*102*	*4*	*4*
*Total severe + shock patients*		*322*	*263*	*59*	*18*
***Total classification 1992 = 2003***		***412***	***348***	***64***	***16***

**Mismatching classification 1992 > 2003**					

**Septic shock**	Severe sepsis	2	2	0	**0**
**Septic shock**	Sepsis	1	1	0	**0**
**SIRS shock**	Severe SIRS	4	3	1	33
**SIRS shock**	SIRS	3	2	1	50
Severe SIRS	SIRS	9	8	1	13
SIRS	No infection	2	1	1	100
***Total shock patients 1992***		***10***	***8***	***2***	***20***
*Total severe patients 1992*		*9*	*8*	*1*	*11*
*Total severe + shock patients 1992*		*19*	*16*	*3*	*16*
***Total mismatching classification 1992 > 2003***		***21***	***17***	***4***	***19***

**Mismatching classification 2003 > 1992**					

Severe sepsis	**Septic shock**	22	21	1	**5**
Sepsis	**Septic shock**	18	17	1	**6**
Infection	**Septic shock**	6	6	0	**0**
Severe SIRS	**SIRS shock**	16	13	3	**23**
SIRS	**SIRS shock**	42	39	3	**8**
No infection	**SIRS shock**	8	8	0	**0**
Sepsis	Severe sepsis	19	18	1	6
Infection	Severe sepsis	6	6	0	0
SIRS	Severe SIRS	71	71	0	0
No infection	Severe SIRS	44	44	0	0
Infection	Sepsis	1	1	0	0
No infection	SIRS	56	56	0	0
***Total shock patients 2003***		***112***	***104***	***8***	***7***
*Total severe patients 2003*		*140*	*139*	*1*	*1*
*Total severe + shock patients 2003*		*252*	*243*	*9*	*4*
***Total mismatching classification 2003 > 1992***		***309***	***300***	***9***	***3***

**Total classification 1992 and/or 2003**					

***Total shock patients 1992 and/or 2003***		***338***	***273***	***65***	***19***
**Total patients**		**742**	**665**	**77**	**10**

## Discussion

The present study mainly proposes that conclusions drawn from data where severity of sepsis is analysed by the 1992 or the 2003 sets of definitions may vary and have to be interpreted with care because the case mix will be different. Interestingly, within the same collective of postoperative/posttraumatic patients, the prevalence of severe sepsis and septic shock was higher when the original 1992 ACCP/SCCM sepsis definitions were replaced by the revised 2003 SCCM/ESICM/ACCP/ATS/SIS sepsis definitions. As a consequence, use of the 2003 definitions was associated with a lower mortality rate in patients with septic shock. The 2003 definitions predicted fatal outcome with slightly higher sensitivity and lower specificity than the 1992 definitions. Patients under-classified by the 1992 definitions, are those who might benefit from a classification into a higher severity state by a switch to the 2003 definitions, leading to earlier and more appropriate critical care management.

To assess potential clinical consequences for surgical patients, at least three aspects (diagnosis, therapy and clinical studies) should be taken into account.

In the revised 2003 definitions, facilitating a bedside diagnosis of sepsis had primacy over research entry criteria [3,4]. The revised 2003 SCCM/ESICM/ACCP/ATS/SIS sepsis definitions were constructed to detect more cases of SIRS/sepsis. As shown in the present paper, this aim has been achieved, since out of the 742 surgical patients, the numbers of no SIRS/no sepsis patients have been decreased by 119 patients with the 2003 definitions. Using the revised 2003 definitions, the increase in prevalence of sepsis was most pronounced in the more severe stages, i.e. in severe sepsis and septic shock (Table 2). Thereby, within the same population, about 27% more surgical patients were allocated to septic shock than they would have been using the 1992 definitions. Thus, applying the 2003 definitions instead of the 1992 definitions will create a case mix with more patients with sepsis, especially classified as more severely ill, i.e. with severe sepsis and septic shock. When comparing classification before vs. after the ACCP/SCCM conference, 16% (n = 57 studies) vs. 3% (n = 119 studies) explicitly required blood culture positivity as an inclusion criterion, respectively [1]. Only 49% vs. 68% of the studies before vs. after the ACCP/SCCM conference incorporated markers of organ dysfunctions into the inclusion criteria [1]. This indicates a trend to prefer criteria of host response (and organ dysfunction) vs. infection diagnostics for the definition of sepsis. Using the 1992 definitions, a threshold of greater than two points in one organ system in the SOFA score has been recommeded to assign organ dysfunction [5]. The higher prevalence of more severely ill classified patients with the 2003 definitions in the present study is due to a lower threshold to define organ dysfunction (in comparison equal to or less than two points in the SOFA score) with the 2003 definitions (Table 1).

Regarding therapeutic approaches, more surgical patients with septic shock are expected to be recommended for potential immunomodulatory treatment using the 2003 definitions. In 2004 and 2008, the "Surviving Sepsis Campaign" guidelines for the management of severe sepsis and septic shock were published to improve outcome [6,7]. Based on the increased prevalence of severe sepsis and septic shock in the present study, more patients may be applicable to and may benefit from the "Surviving Sepsis Campaign" management guidelines. On the other hand, this may lead to more undesirable consequences, such as harm to patients, more burden on staff and patients, and greater costs. One of the evidence based recommendations suggests the application of recombinant human activated protein C (rhAPC) in patients at high risk of death (Acute Physiology and Chronic Health Evaluation II, APACHE II >= 25 and septic shock, as defined by the 1992 definitions), since rhAPC has been shown to reduce mortality in these patients by 13% [6,7]. Applying the 2003 definitions, in our ICU, within one year, 43 more surgical patients, i.e. about 21% more patients, would have been potential candidates for this expensive and potentially harmful (risk of bleeding) therapy. Moreover, their mortality rate of 22%, in any case, would have been markedly lower than with the 1992 definitions (Table 2). Thus, it may be necessary to retrospectively or prospectively revaluate whether treatment recommendations based on the 1992 definitions hold true, especially, if the same population is classified with the 2003 definitions.

With respect to inclusion criteria for sepsis trials, the present study revealed that more surgical patients with septic shock, having a lower mortality rate, would have been enrolled using the 2003 definitions (Table 2). Thus, a properly powered study with the 2003 definitions would have to be larger, in order to demonstrate an equivalent mortality reduction (by e.g. 10%) compared with the same population classified by the 1992 definitions in which the mortality rate is higher.

Since sepsis definitions lack specificity and sensitivity, efforts have been made [3,4,9] and will be mandatory in the future to improve diagnosis and valid classification of patients to reduce variability within treatment groups, and to increase the probability of generating convincing results. In the present study in critically ill surgical patients, prediction of death by the 2003 definitions occurred with slightly higher sensitivity and lower specificity than by the 1992 definitions. Replacing the original 1992 sepsis definitions with the 2003 revised sepsis definitions resulted in a different case mix with higher prevalence of severe sepsis and septic shock. Consequently, mortality rates of patients with septic shock were lower. In the future, it has to be clarified whether, in addition to clinical scores, well defined immune parameters, such as distinct biomarker profiles, may improve diagnosis of infection and severity of disease, prediction of outcome, guidance and success of therapeutic interventions [10]. Predisposing factors for sepsis have been reported to occur in 20% of all sepsis trials between 1976 and 2001 [1]. Thus, the revised 2003 SCCM/ESICM/ACCP/ATS/SIS sepsis definitions conference introduced the PIRO model, i.e., predisposition, infection/insult, response and organ dysfunction, as a staging system for sepsis [3,4]. Moreover, to increase uniformity in patient enrolment, comprehensive demographic data including severity scores, such as the SAPS II [8] or SAPS 3 [11] to assess severity of illness and predict vital status at hospital discharge, will be required.

Regarding divergent classifications of patients, the present study shows that 309/742 patients were under-classified with the 1992 definitions, but 21, only, with the 2003 definitions (Table 4). Also, in the patients with severe SIRS and sepsis, and shock, markedly more patients were under-classified with the 1992 definitions as compared to the 2003 definitions. Especially these patients with high risk of death under-classified by the 1992 definitions are those who might benefit from earlier and more appropriate critical care management, if, in addition, detected by the 2003 definitions.

## Conclusion

The present study shows that the definition of sepsis criteria strongly influences prevalence and mortality rates of sepsis. Use of the 1992 definitions may lead to under-classification of patients with severe sepsis at high risk of death with the consequence of delayed or lack of timely and appropriate critical care management. Thus, the present study underlines the need for a widespread use of common sepsis definitions to facilitate comparability, diagnosis, treatment recommendations, and enrolment strategies for clinical trials of surgical patients with sepsis. Due to the fact that only surgical patients have been enrolled in the present study, it has to be clarified whether the results are applicable to medical cases, also.

## Key messages

Applying the 2003 SCCM/ESICM/ACCP/ATS/SIS sepsis definitions instead of the 1992 ACCP/SCCM sepsis definitions in surgical patients resulted in different patient case mix with under-classification with the 1992 definitions.

Within the same surgical patient collective, applying the 2003 definitions instead of the 1992 definitions, prevalence of severe sepsis and septic shock was higher.

In surgical patients with septic shock, mortality rate was lower using the 2003 definitions than with the 1992 definitions.

Risk of death was increased for those surgical patients classified to be in septic shock in both definitions compared to those classified to be without septic shock in both definitions (OR 6.5, p = 0.001).

The 2003 definitions predicted fatal outcome in surgical patients with slightly higher sensitivity and lower specificity than the 1992 definitions.

## Abbreviations

ACCP/SCCM: American College of Chest Physicians/Society of Critical Care Medicine; APACHE II: Acute Physiology And Chronic Health Evaluation; 95% CI: 95% confidence interval; CI: confidence interval; ICU: intensive care unit; IR: Inflammatory Response Syndrome; INR: International Normalized Ratio; OR: odds ratio; n. a.: not applicable; PaO_2_/FiO_2_: partial pressure of arterial oxygen/fraction of inspired oxygen; SAPSII: Simplified Acute Physiology Score; SCCM/ESICM/ACCP/ATS/SIS: Society of Critical Care Medicine/European Society of Critical Care Medicine/American College of Chest Physicians/American Thoracic Society, Surgical Infection Society; SD: standard deviation; SIRS: Systemic Inflammatory Response Syndrome; SOFA: Sequential Organ Failure Assessment Score; SvO_2_: mixed venous oxygen saturation; WBC: white blood cells.

## Competing interests

The authors declare that they have no competing interests.

## Authors' contributions

MW, MHL, MK, BH and MS participated in study conception, study design, data analysis, interpretation and drafting of the manuscript. MW, KT and JA participated in data acquisition, data analysis, and interpretation of the manuscript. MT, MW and JA participated in programming the computer-assisted scoring systems and data base, data analysis and interpretation of the manuscript. All authors read and approved the final manuscript.

## Pre-publication history

The pre-publication history for this paper can be accessed here:

http://www.biomedcentral.com/1472-6947/9/25/prepub
